# Operationalizing digital twins in biomanufacturing through interoperable process analytical technology

**DOI:** 10.1007/s00449-026-03369-9

**Published:** 2026-07-16

**Authors:** Abhijeet Satwekar, M. Cyndell Gracieux-Singleton, Chris Cummings, Jonquil A. R. Horton, Monica Accerbi, Veerabhadraiah Palakollu, Ryan R. Barton, Sandeep Kedia, Thomas Cornish, Katharina S. Yandrofski, Roger Hart, Dannielle Berlinghieri, James Saylor, Kelvin H. Lee

**Affiliations:** 1 Analytical Excellence & Program Management, Global Analytical Development, Global CMC Development, Merck Serono S.P.A. (A Business of Merck KGaA, Darmstadt, Germany), Via Luigi Einaudi 11, Guidonia Montecelio, 00012, Rome, Italy; 2 Golden LEAF Biomanufacturing Training and Education Center, North Carolina State University, 850 Oval Drive, Raleigh, NC 27606 USA; 3 Technical Operations, National Institute for Innovation in Manufacturing Biopharmaceuticals, 590 Avenue 1743, Newark, DE 19713 USA; 4 National Institute for Innovation in Manufacturing Biopharmaceuticals, 590 Avenue 1743, Newark, DE 19713 USA; 5 Global Technology, Engineering, & Launch, Pfizer Inc, 7000 Portage Rd, Kalamazoo, MI 49001 USA; 6 Institute for Bioscience and Biotechnology Research, National Institute of Standards and Technology, 9600 Gudelsky Drive, Rockville, MD 20850 USA; 7 Project Management Office, National Institute for Innovation in Manufacturing Biopharmaceuticals, 590 Avenue 1743, Newark, DE 19713 USA

**Keywords:** Process analytical technologies, Digital twins, Interoperability, Model-based predictive control, Biomanufacturing

## Abstract

The integration of Process Analytical Technologies (PAT) within digital twin architecture represents an important advancement in biopharmaceutical manufacturing. While existing literature has separately addressed digital twin dimensions, interoperability levels, and manufacturing standards, their systematic integration for real-time bioprocess control remains underexplored. Our work provides an operational framework that bridges established frameworks on digital twin dimensions with hierarchical interoperability levels. Our conceptual framework establishes an interoperability “triangle” wherein Data and Connection dimensions function as the central hub linking Physical and Virtual entities with Services to enable model-based predictive control (MPC) through PAT integration. We exemplify the practical application of our proposed approach using a galactosylation adaptive control case study that spans purpose-driven systematic technology assessment to operational deployment at the testbed. Our study provides theoretical advancement in digital twin operationalization and a practical approach for implementing MPC-enabled PAT systems in highly regulated biopharmaceutical manufacturing, with potential for applicability across multiple critical quality attributes, unit operations, and manufacturing scales.

**Supplementary Information:**

The online version contains supplementary material available at 10.1007/s00449-026-03369-9.

## Introduction

Process Analytical Technologies (PAT) have become indispensable for real-time monitoring and control of critical quality attributes (CQAs) and process parameters in biopharmaceutical manufacturing [1, 2]. The integration of PAT facilitates a Quality by Design (QbD) framework, enabling a comprehensive understanding and control of bioprocesses to ensure consistent product quality and enable real-time release testing [3, 4]. Further advancements in PAT implementation have focused on integrating model-based strategies and novel analytical technologies to enhance process control and optimization. The use of in-line analytical tools, such as index of refraction (IoR) sensors, has been demonstrated for real-time protein concentration measurement during downstream operations, contributing to an improved continuous bioprocess manufacturing [5]. The integration of model-based design of experiments (DoE) with PAT has shown potential in addressing challenges associated with bioprocess scale-down and scale-up, and enabling more accurate representation of industrial-scale processes at the laboratory scale [6]. Quantitative mass spectrometry (MS), particularly liquid chromatography-mass spectrometry (LC–MS) has expanded its applications beyond routine characterization to include process development and manufacturing as a PAT application to provide information on product quality attributes and impurities [7]. Raman spectroscopy as a PAT facilitates advanced process control and enables consistent product quality. The applications based on Raman spectroscopy range from single-cell analysis to downstream process monitoring and have prospective value throughout a biopharmaceutical product’s lifecycle [8]. Machine learning approaches have been proposed to improve the calibration of the generic Raman models for real-time monitoring of cell culture performance parameters ranging from glucose, glutamate, glutamine, ammonium, lactate, sodium, calcium, viability, and viable cell density [9]. Innovations in sensor technologies are pivotal for the monitoring and control of biomanufacturing processes. The implementation of robust sensor systems and chemometric techniques facilitates continuous real-time monitoring, contributing to more stable, reproducible, and efficient processes. Advanced sensor systems, such as near-infrared (NIR) spectroscopy, provide real-time, non-invasive measurements of critical process variables to enable improved control [10, 11]. Despite their potential, technologies like NIR spectroscopy are not yet widely applied in biomanufacturing (Table 1).

**Table 1:** Interoperability for PAT for model based predictive control

Digital Twin Framework—Dimensions	Functions	Interoperability principles	Interoperable characteristics for PAT to enable adaptive control
Physical Entities	Generate data and provide control	Devices are connected, controlled, and generate data with seamless integration with data management and process control systems	Devices from sampling, sample preparation, analytical systems, sensors and controllers are seamlessly integrated and controlled with a feedback loop
Virtual Entities	Digital representation for physical entities e.g. models, simulations	Systems provide exchange between the physical and virtual entities, allowing data processing and analytics for virtual models to simulate and predict the desired outcomes	Systems provide real-time data to virtual models for analytics, and perform predictive modelling, virtual simulation to inform adaptive control strategies
Data	Information representations	Information is exchanged across the different systems in a consistent and standard format, to enable decision making Data management, traceability and interconnectivity by bridging the historical and evolving data to keep the contextual relevance	Information exchange is accurate, timely, fit-for-purpose and can drive informed decisions and process adjustments. Data is managed and ensured its availability for audits
Connection	Communication & interconnections between entities	Robust and widely compatible common communication protocols that ensure efficient data exchange between different systems, allowing for scalability and flexibility in adapting to new technologies	Systems are interconnected using standardized protocols to ensure efficient and real-time communication between PAT tools and control systems enabling plug and play
Services	Outcomes from the digital twin system	Integrate and manage various functionalities, such as task execution, monitoring, analysis, and predictive insights to trigger controllers towards a desired outcome	Systems interexchange and operate to facilitate monitoring, analysis, and predictive modelling to derive actionable insights for triggering the control adjustments for optimizing operations, quality and responsiveness

In continuous bioprocessing, model-based real-time control strategies, such as mechanistic models for pH adjustment between capture chromatography and viral inactivation steps, have been developed to achieve tight control of critical parameters and ensure product quality [12]. Furthermore, online optimization techniques utilizing Advanced Process Control (APC), such as model predictive control (MPC) have been applied to capture chromatography step to optimize dynamic binding capacity and productivity—demonstrating the benefit of combining soft sensors and predictive modeling within PAT frameworks [13]. In the upstream unit operation, the applications for model-based control are reported for yield, glycosylation, glucose, cell and media components. These applications may utilize mechanistic, data-driven, and hybrid model-based control techniques. Recent studies have showcased the benefits in monoclonal antibody (mAb) production. A feeding strategy was optimized via MPC to maximize cell growth and metabolite production in fed-batch bioprocess, that resulted in more than 2% improvement in final protein production, as compared to the average historical experiments [14]. A hybrid model combining mechanistic mass balances with data-driven random forest regression was developed to enable accurate prediction of mAb and impurity concentrations under varying conditions and optimize the cultivation for maximum yield and quality [15]. An MPC strategy was applied using a hybrid kinetic-stoichiometric model for CHO cell cultures, achieving up to 95% higher antibody production and demonstrated a real-time, transferable feeding optimization [16]. These applications demonstrate the value of model-based strategies to enable both offline process optimization and real-time control, leading to higher productivity, improved product quality, and operational efficiency in mAb biomanufacturing.

Given the complexity of biopharmaceutical manufacturing and the increasing interest in real-time process optimization and control, the establishment of digital twins (DT) is paramount. Digital twins are virtual replicas of physical systems that continuously exchange data, for monitoring, prediction, and control decision-making. Digital twins facilitate a robust bioprocess design, enabling virtual testing and optimization before implementation in the physical process, thus reducing the need for extensive exploratory experiments and costs [17]. Recent developments in computational fluid dynamics (CFD) and stimulus–response metabolic models have improved process prediction and evaluation, enabling faster scale-up with minimal performance losses [18]. For instance, an automated end-to-end process integrating a perfusion bioreactor with downstream purification steps for antibody production was demonstrated as a continuous manufacturing platform [19]. In this case, a Supervisory Control and Data Acquisition (SCADA) system was developed for digital integration of unit operations, enabling centralized data collection, process monitoring, and control. This SCADA system adjusted process parameters in real-time in response to disturbances, maintaining optimal performance despite decreasing cell-specific productivity [19]. The effectiveness of a digital twin system relies on how information or data is organized in detail at various levels and how it is interconnected. Data interoperability achieved through standardized data formats, common semantic frameworks, and compatible communication protocols facilitates the reduction of silos, and provides seamless integration of heterogeneous data sources from sensors, control systems, and analytical platforms. While existing literature has separately addressed digital twin dimensions [20], interoperability levels [21], and digital twin standards [22], their operational integration remains underexplored. Our work addresses this gap by bridging the established frameworks on digital twin dimensions [20], and interoperability levels [21] into a merged operational framework for practical implementation. We present a case study on glycan adaptive control to illustrate the practical implementation of interoperability components across technical, syntactic, semantic, pragmatic, dynamic, and organizational levels, merged with the digital twin dimension framework. We demonstrate the practical application of our proposed merged framework, offering general guidelines for advancing digital twins in biomanufacturing while enabling real-time process control and optimization capabilities.

### Conceptual framework for interoperable digital twin

#### Digital twin dimensions and interoperability for PAT

A 5-dimensional digital twin standard framework model was introduced for developing digital twin (DT) applications. These dimensions consist of Physical Entities, Virtual Entities, Data, Connection, and Services [20]. The Physical Entities dimension emphasizes integrating real-world physical systems into DT environments, ensuring seamless data exchange between physical devices and their virtual counterparts to perform the necessary valuable services. The Virtual Entities dimension includes the virtual models that accurately reflect the dynamics and characteristics of their physical counterparts and support the prediction of necessary inputs based on the data. The Data dimension emphasizes the data exchange between the physical, virtual and services dimensions. It focuses on ensuring that data is consistent, reliable, accurate, and organized at an increasing level of depth. The Connection dimension establishes robust communication protocols for connecting all the dimensions to ensure efficient data exchange and connectivity within digital twin environment. Finally, the Services dimension outlines the essential services and functionalities that digital twins are expected to provide, including execution tasks, monitoring, analysis, predictive maintenance, service integration and management to effectively oversee and interpret data to optimize operations. A comprehensive detailed list of specific standards under each dimension of the digital twin framework is elaborated in a recent review [20].

**Fig. 1:**
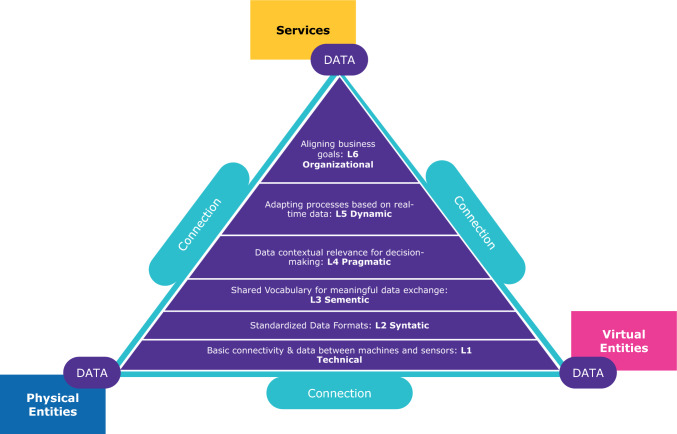
Interoperability “Triangle” connecting the digital twin dimensions

Process Analytical Technologies (PATs) for process monitoring and Model-Based Predictive Control (MPC) requires seamless exchange of information. Requirements for a PAT application include, a) Real-time data acquisition within the -desired frequency and speed, b) end-to-end integration with data management and process control systems, e.g. Distributed Control Systems (DCS), Supervisory Control and Data Acquisition (SCADA) systems, and compatibility with the MPC architecture for the exchange of data with controller, c) Analytical data processing and generating results in real-time, d) Intuitive user interface to monitor the analyte signals, e) Scalability and flexibility to connect and adapt with evolving instrumentations and technology, f) Fit-for-purpose data to enable actions, ensuring that the data from PAT application is relevant and actionable, and g) Compliance with current regulatory standards and guidelines. Collectively, these requirements underscore the need for an interoperable environment that ensures seamless data exchange and organized information management across all levels of PAT application. Interoperability is defined as “the ability of two or more systems or components to exchange information and to use the information that has been exchanged” [23, 24]. Interoperability is an integral element for integrating various technologies into the digital twin application. An interoperability framework is proposed for digital twins that is hierarchically organized into six levels—Technical, Syntactic, Semantic, Pragmatic, Dynamic, and Organizational [21]. The first level, Technical, focuses on basic connectivity and data exchange among physical assets, sensors, actuators, and related components. The second level, Syntactic, emphasizes the use of standardized data formats and protocols to ensure uniform data structures and facilitates standard data exchange. The third level, Semantic, centers on standardized ontologies and contextual metadata to achieve uniformity and enable meaningful data exchange across systems. The fourth level, Pragmatic, addresses the contextual use of data in operational processes, tailoring actions to contextual insights and supporting actionable solutions. The fifth level, Dynamic, introduces adaptability by enabling interoperability amid evolving conditions and requirements, thus supporting adaptation to changes in data, context, or processes. Finally, the sixth level, Organizational, seeks alignment with business objectives and policies, promoting system adaptability, ecosystem coherence, and integrated operations [21]. To design an effective digital twin application, the five-dimensional digital twin framework must be integrated with the six-level interoperability framework, creating a structural system representation, with organized data interoperability that facilitates seamless information exchange and drives desired operational actions. The five dimensions of Physical Entities, Virtual Entities, Data, Connection, and Services, provide the foundational architecture for digital twins. Meanwhile, the six-level interoperability framework facilitates data standardization, seamless information exchange, semantic consistency, contextual understanding, adaptability, and organizational alignment across these dimensions. At the Technical and Syntactic levels, the Physical Entities and Connection dimensions must be supported by standardized hardware interfaces, communication protocols, and data formats, ensuring reliable connectivity and data exchange. The Semantic level requires the Data and Virtual Entities dimensions to adopt standardized ontologies and metadata schemas, enabling meaningful interpretation and integration of information across diverse systems. The Pragmatic and Dynamic levels within the Services dimension entails context-aware analytics, real-time decision-making, and adaptive process control, thus enabling the digital twin to respond intelligently to evolving operational scenarios. Finally, the Organizational level is achieved when all five dimensions are harmonized with business objectives that are focused on achieving value drivers e.g. efficiency, quality, compliance etc. The Data and Connection dimensions play a central role in linking Physical Entities, Virtual Entities, and Services, forming a “triangle” of interoperability levels organized around connectivity and data. This represents a merged framework for a digital twin application (Fig. 1). Furthermore, ISO 23247, the international standard for "Automation Systems and Integration—Digital Twin Framework for Manufacturing," establishes overarching principles, functional reference models, information representation methods, and data exchange protocols for digital twin implementation in manufacturing applications [22]. ISO 23247 standard enhances the five-dimensional digital twin framework by providing standardized components, methods, and interfaces for secure integration and communication across interoperability levels. ISO 23247 is particularly valuable for highly regulated biopharmaceutical environments, enabling regulatory compliance, lab-to-production scalability, seamless vendor interoperability, data integrity, and structured risk management.

#### Technology assessment framework

The selection of Process Analytical Technologies (PAT) is a critical determinant for a digital twin in biomanufacturing. PAT generates data at the required frequency, accuracy, and semantic depth aligned with the intended context of use. Within the digital twin applications, the PAT selection must be guided not only by analytical capability, but by its ability to generate interoperable, decision-enabling data that can be seamlessly integrated across the digital twin dimensions to enable meaningful actions for value generation. We propose a structured systematic technology assessment framework, which consists of three sequential steps: [1] definition of the analytical and operational objective through an Analytical Target Profile (ATP): establish the intended purpose, critical quality attribute (CQA), performance requirements, and decision time window; (2) identification of candidate analytical technologies: capable of meeting the decision window and information depth required; (3) systematic evaluation of the analytical technologies: using a multi-criteria assessment rubric encompassing performance, technical readiness, risk, and business value.

A comprehensive evaluation rubric serves as a critical tool for technology assessment, enabling systematic evaluation of technological capabilities and their alignment with operational, business needs and value. The framework comprises four equally weighted categories: performance, technical, risk, and business value, each subdivided into specific sub-categories detailed in Table 2. Towards informed decision-making, the sub-categories are assessed using a three-point Likert scale (5, 3, 1) for a detailed technology comparative analysis. The first performance category is crucial for determining how well a technology meets the operational criteria, and it includes four key metrics: specificity, linearity of response, accuracy and precision, each weighed equally at 0.25. Specificity is critical in PAT as it determines the ability of the technology to identify and measure specific desired components or attributes. High specificity ensures that technology can target and analyze the desired analytes without interference from other components, leading to more reliable and meaningful data. Specificity is assessed from high (score 5) to low (score 1), focused for targeted analytes. Linearity of response refers to the ability of the technology to produce results that are directly proportional to the concentration of the analyte across a specified range. This is crucial for ensuring that the PAT can accurately track changes and maintain consistency in measurements, which is essential for process control and optimization. Scoring for Linearity of response ranges from quantitative (score 5) to qualitative/classification-based outcome (score 1), where the quantitative measures provide more informed data interpretation. Accuracy is a measure of how close the results obtained by technology are to the true value, i.e. theoretical value or comparison to the established analytical reference. High accuracy is essential to ensure that the data generated by PAT is reliable and can be used for critical process decisions. Scoring for Accuracy is categorized into three levels: 91–100% (score 5), 80–90% (score 3), and below 80% (score 1). Precision refers to the consistency and reproducibility of analytical results. High precision ensures that repeated measurements under identical conditions yield consistent results. This is critical for bioprocess monitoring and control, where measurement variability can lead to significant process deviations. Scoring for Precision is evaluated based on the coefficient of variation (CV) with a score of 5 assigned for CV less than 10%, a score of 3 for CV between 11–25%, and a score of 1 for CV above 25%. The second technical category is assessed through two sub-categories, each weighed at 0.5. The sub-category of readiness assesses the maturity of the technology following the Biomanufacturing Readiness Level (BRL) scale [25], with BRL 7–9 (score 5) for technologies that are pilot validated and proven for commercial use, to BRL 1–3 (score 1) for the technologies at the idea or proof of concept stage. It is important to determine whether PAT technology is sufficiently matured for its integration into the routine operations in development and good manufacturing practices (GMP) environments. The set-up sub-category was chosen to assess the applicability of the analytics as online/in-line (score 5), at-line (score 3), or offline (score 1). The deployment of PAT (online/in-line, at-line, or offline) affects how data is collected and integrated into the operations. Online/in-line setups provide more frequent real-time data, which is most desired for immediate process adjustments and maintaining control over critical parameters. Although, the time window necessary for decision making can further influence the choice between online/in-line and at-line. The third Risk category consists of four sub-categories, each weighing 0.25. The sub-category Stability and Obsolescence relate to the longevity and relevance of technology. Technologies that remain stable and relevant over time are more likely to provide consistent data and reduce the need for frequent updates or replacements, which can be costly and disruptive. Therefore, we assigned a score of 5 for technologies that would last more than 10 years, while those soon to be obsolete with a score of 1. Sub-category of Fluidity and Customizability refers to the technology’s ability to adapt to changes or to have compatibility with legacy systems of the organizations. High fluidity allows for greater flexibility in application, enabling the technology to meet evolving process needs and integrate with other systems. Thus, high fluidity is indicated with a score of 5 and low fluidity with a score of 1. The sub-category on User Requirement Specification (URS) assesses the necessary features and capabilities that technology must possess to meet the specific needs of the desired operational process. The User Requirement Specification (URS) is evaluated, with full alignments scoring 5 and partial alignments with scoring 1. Connectivity and flexibility determine how easily the PAT tool can be integrated with other systems and processes. High connectivity reduces integration efforts and enhances the ability to seamlessly exchange and use data across different platforms. Thus, with low efforts required for integration scoring 5, and high efforts scoring 1. The fourth category of Business Value consists of 4 sub-categories, each weighed at 0.25. Financial assessments consider net present value (NPV) and internal rate of return (IRR), with positive values scoring 5 and negative values scoring 1. Financial assessments by business cases evaluate the economic impact of implementing a PAT system and elaborates an estimation on cost efficiency and return on investment. Understanding the financial implications is needed for justifying the adoption of new technologies and ensuring they contribute tangibly to a positive value. The non-financial sub-category assesses the broader, difficult-to-quantify benefits of PAT, that as they indirectly enhance overall value e.g. (compliance, quality, risk mitigation, cost avoidance etc.). Aligning the PAT assessments with organizational strategies ensures that technology supports broader business objectives. In the context of increasing regulatory and consumer focus on sustainability, such as reducing waste and energy consumption, the PAT tools should support long-term sustainable practices. The sub-category of sustainability evaluates the technology’s contribution to environmental and social impacts as per the Sustainable Development Goals (SDGs) [26]. Therefore, non-financial benefits, strategy alignments, and sustainability sub-categories are evaluated by the number of benefit areas or strategy or SDGs respectively, with more than three (score 5), 2 to 3 (score 3), and 0–1 (score 1) (Table 2).

**Table 2:** Technology Assessment Rubric

Categories	Sub-categories		Score 5	Score 3	Score 1
Performance	Specificity	0.25	High	Medium	Low
	Linearity—response	0.25	Quantitative	Semi-quantitative	Qualitative/classification
	Accuracy	0.25	91–100%	80 to 90%	Below 80%
	Precision	0.25	Less than 10% CV	11 to 25% CV	Above 25% CV
Technical	Readiness	0.5	BRL 7 to 9: pilot validated & proven for commercial	BRL 4 to 6: Early to late prototype tested	BRL 1 to 3: Idea/Proof of concept
	Set-up	0.5	Online/In-line	At-line	Offline
Risk	Stability—Obsolescence	0.25	more than 10 years	5 to 9 years	soon to be obsolete
	Fluidity—customizability	0.25	high fluidity	medium fluidity	low fluidity
	URS	0.25	Fully met	partially met	needs adaptation
	Connectivity /Flexibility	0.25	low efforts	medium efforts	high efforts
Business Value	Financial	0.25	positive (NPV, IRR)	N.A	negative (NPV, IRR)
	Non-financial	0.25	more than 3 benefit area	2 to 3 benefit area	1 benefit area
	Strategy alignments	0.25	more than 3 strategies	aligned to 2 to 3 strategies	aligned to 0 to 1 strategy
	Sustainability	0.25	more than 3 SDG	2 to 3 SDG	0–1 SDG

#### Use case instantization

Glycans at the Fc region of the monoclonal antibody are responsible for Fc mediated effector functions for clinical activity and influence the pharmacokinetic and pharmacodynamic behavior, while some glycan structures are also involved in adverse immune reactions [27]. From a manufacturing perspective, glycosylation is particularly challenging to control because of its high sensitivity to upstream process conditions. Often, the processes exhibit run-to-run glycan variability, which shifts the glycan profile, affecting the product quality and potentially its clinical performance. The presence or absence of galactose at the terminal end of glycan modulates majorly the Complement-dependent cytotoxicity (CDC), and minorly influences the Antibody-dependent cellular cytotoxicity (ADCC), and Antibody-dependent cell-mediated phagocytosis (ADCP) with a dependency on a-fucosylation levels [28]. Beyond effector function, galactosylation influences Fc region thermal stability, FcγR binding affinity, and serum half-life, making it an important quality attribute with both safety and efficacy implications. Even for the biosimilar developers, comparability needs to be demonstrated for the galactosylation profile with the reference product. Therefore, we chose galactosylation as target Critical Quality Attribute (CQA) for the demonstration of adaptive control within the BTEC SPIDER (Sensors and Software for Process Control, Integration, Data Analysis, Process Evolution, and Reporting) Testbed (BTEC Testbed). Adaptive control of galactosylation represents a use case with direct translational relevance to both innovator and biosimilar biomanufacturing, for maintaining a defined galactosylation distribution in real time and generating a consistent, homogeneous product with a desired quality profile. For the meaningful operational demonstration of the use case, we selected NIST reference monoclonal antibody standard (NISTmAb, RM 8671) rather than a proprietary therapeutic molecule, as it is a well-characterized molecule with a defined and publicly available glycan profile. Galactosylation was selected as the sole target attribute for this demonstration to simplify the elaboration of the framework and focus the proof-of-concept on a single quality attribute. This simplification does not reflect a limitation of the approach and can be directly extended to other glycan attributes such as fucosylation, high-mannose content, sialylation, and afucosylation, depending on the product specific quality requirements. The use case is presented for demonstration purposes only and does not relate to any marketed product. A model-based glycosylation prediction and control requires data from multiple at-line sensing analytical technologies, as input variables to provide outcomes for process adjustments (Supplementary Table 1). Within the scope of this article, we demonstrate the instantiation of the most analytically complex component of this system, i.e. glycosylation distribution, which provides critical analytical data required by the digital twin architecture. Establishing the glycan analytics component end-to-end, from method development through at-line integration and adaptive control demonstration, is the central contribution of this work, and provides the foundation for building the full system integration around all the input variables.

## Materials and methods

### Subunit method (lab bench– offline analytics) – BTEC testbed

Lyophilized monoclonal antibody (mAb) subunit standard (Waters, P/N 186008927) was used for method development. The lyophilized protein was reconstituted with 10 mM Tris Buffer (pH 7.3) to 1 mg/mL concentration. For the samples from the bioreactor, Protein A affinity purification was performed with Thermo Scientific spin plates (Pierce). The manufacturer’s protocol was followed, with minor modification of the neutralization step to include the IdeS digestion buffer to ensure the conditions were suitable for efficient IdeS digestion. IdeS protease at a ratio of 1 U enzyme per 1 µg protein was added to the mAb standard or mAb bioreactor sample and incubated at 45 °C for 30 min. Dithiothreitol (DTT) was added at a final concentration of 100 mM and incubated at 45 °C for an additional 30 min. To accommodate the variable mAb titers observed across different bioreactor sampling days, a standardized IdeS addition protocol was implemented. A constant enzyme volume was added based on the maximum expected titer and applied consistently to all samples, thereby eliminating the requirement for individual titer determination at each sampling timepoint while ensuring adequate enzymatic digestion. The resulting subunit samples were subsequently analyzed using liquid chromatography (LC) or liquid chromatography-mass spectrometry (LC–MS) approaches.

### Subunit method—chromatographic and mass spectrometric analysis – BTEC testbed

At the BTEC Testbed, the analysis was performed on an ACQUITY UPLC system (binary pump; flow-through needle (FTN)) coupled to a time-of-flight mass spectrometer (BioAccord, Waters). Chromatographic separation of the mAb subunits was performed on a BEH Amide Hydrophilic Interaction Liquid Chromatography (HILIC) column (Waters), maintained at 45 °C, with a guard column installed upstream of the analytical column. Mobile phase A consisted of 0.1% trifluoroacetic acid (TFA) in water, and mobile phase B consisted of 0.1% TFA in acetonitrile. The chromatographic separation was performed with 0.4 ml/min flowrate on an ACQUITY UPLC Glycoprotein BEH Amide column (300 Å, 1.7 μm, 2.1 × 150 mm, Waters #176,003,702), and using the starting gradient conditions of 28% of mobile phase A. Gradient was linearly increased to 36% in 10 min, and then 70% in 0.1 min, with a hold time of 2 min. The column was re-equilibrated with 28% mobile phase A within 0.2 min, and held for 4 min. The fluorescence detection parameters were 280 nm and 360 nm for the excitation and emission wavelengths. The mass spectrometer was operated in positive electrospray ionization mode with a capillary voltage of 1.5 kV, cone voltage of 120 V, and desolvation temperature set to 550 °C. Mass spectra were acquired over a mass range of 400 to 7000 m/z at a scan rate of 2 Hz. The initial eluent flow was diverted to waste for the first 2 min to prevent the introduction of non-volatile salts into the mass spectrometer. Data processing was performed using UNIFI software (Waters), applying automatic integration and optimized shoulder peak detection. MS data were further processed using the MaxEnt1 deconvolution algorithm.

### Subunit method – fit for purpose performance – NIIMBL testbed

The sample preparation and chromatographic analysis procedure from BTEC Testbed was adopted at NIIMBL Testbed with minor changes for lab instrumentation. The bioreactor sample from 14th day was used to assess the method performance using the SLAP (specificity, linearity, accuracy, and precision) approach for determining it to be fit for purpose towards galactosylation monitoring. The LC subunit method was performed using Waters UPLC Instrumentation (Waters Acquity Premier: Sample Manager FTN and Quaternary Solvent Manager; PDA eλ Detector). Specificity was assessed by analysis of two samples as, a) containing the mAb sample, and b) did not contain the mAb sample. Both samples were treated with same sample preparation and chromatographic analysis. The evaluation of specificity was performed by observation of the absence of peaks in the non-mAb sample, and presence of peaks in the mAb-containing sample. Linearity of response for individual glycan species was evaluated across five concentration levels (50%, 75%, 100%, 125%, and 150%) corresponding to the load amount of 0.5–1.5 µg of sample quantity. At each level, three independent sample preparations were analyzed, and the chromatographic peak area attributable to each glycan species was integrated. Linearity was determined from the observed peak areas versus loading levels and statistically evaluated for linear regression: coefficient of determination (R^2^), for each glycan species to assess goodness of fit. Accuracy was assessed at three nominal load levels (50%, 100%, and 150 in triplicates, and the relative distribution (%) of the observed glycan species was determined. Accuracy for each glycan species was expressed as percent recovery, calculated as (observed value/theoretical value) × 100. Precision was evaluated at the 100% target loading (1 µg). Repeatability was determined from six independent sample preparations analyzed on Day 1 by Operator 1. Intermediate precision was determined from six additional independent preparations analyzed on Day 2 by Operator 2. A variance components analysis was performed to estimate the variance (day/operator), and results were reported as percent relative standard deviation (%RSD). The DOE samples were analyzed by using 100% target loading concentration and reported as relative distribution of glycan species.

### Offline to at-line glycan monitoring configuration

Sub-unit glycan method was adapted from the offline lab bench to the bioreactor at-line set-up at the BTEC Testbed (Table 3). The offline method involved the processing of clarified bioreactor supernatant through centrifugation and filtration, followed by Protein A spin plate purification. For the at-line set-up, significant instrumental changes were performed. Automated sampling was achieved through a MAST autosampler system. The MAST controllers directed two distinct sample streams from the bioreactor; one cell containing sample was directly transferred to a Nova Biomedical Flex2 BioAnalyzer for analysis of dissolved gases, pH, biochemistry, osmolality, and cell counts and the other was processed through the MAST Cell Removal System (CRS), which is a hollow fiber-based cell separation unit, and then stored in a temperature-controlled rack (4 °C) on a Gilson liquid handler. These cell-free samples underwent automated Protein A purification using 1-mL columns, followed by dilution, IdeS enzymatic digestion and DTT reduction in heated racks, and storage at 4 °C prior to the LC analysis on an ACQUITY UPLC system (Fig. 6). Sample processing was modified from the offline centrifuge-filter approach to utilize the MAST Cell Removal System (CRS) for cell separation, coupled with HiTrap Protein A columns in place of spin plates. The core analytical method remained unchanged, maintaining the 17.2-min chromatographic runtime and IdeS digestion with DTT reduction protocol to ensure method equivalency. Data processing was enhanced from the offline semi-automated FLR-based quantitation with MS confirmation through UNIFI software to an automated workflow utilizing retention time libraries and custom fields within UNIFI. This adaptation removed the manual data processing to achieve end-to-end data transition for real-time application. The bioreactor was equipped with sensors for temperature, pressure, foam level, weight, pH, dissolved oxygen, capacitance-based viable cell density, and off-gas O₂ and CO₂ monitoring. Process control was maintained through mass flow controllers for sparging air, oxygen, and carbon dioxide, along with air overlay capability. The feeding system incorporated gravimetric control for antifoam, base, and two additional feeds via four peristaltic pumps, supplemented by four additional peristaltic pumps equipped with clamp-on flow sensors. The at-line set-up faced several operational constraints that required careful consideration in method adaptation. The Gilson liquid handler operated in single-sample mode, precluding multitasking. Sample processing utilized 12-mL vials necessary to accommodate the required volumes (5–8 mL) for cell-free retains and purified material. Due to the vial style and the need to prevent the Gilson liquid handler’s syringe needle from touching the vial bottom, an inaccessible volume of approximately 200–300 μL was considered. Temperature-controlled racks operated at fixed temperatures with manual control independent of the MAST system, and sample mixing was achieved through syringe arm aspiration cycles.

**Table 3:** Offline to at-line glycan monitoring configuration

Parameter	Offline Workflow	At-Line Monitoring
Sample Type	Clarified Bioreactor Supernatant	5 L Bioreactor Culture broth at-line sample
Instrumentation	Waters UPLC-FLR	MAST Autosampler + Gilson Liquid Handler + Waters UPLC-FLR
Sample Processing	Centrifuge + filter, Protein A spin plates & IdeS digestion + DTT	MAST-CRS, HiTrap Protein A & IdeS digestion + DTT
Chromatography Run Time	17.2 min	17.2 min
Data Processing	FLR-based quantitation (MS confirm), semi-automated (UNIFI)	FLR-based quantitation (RT library), automated via UNIFI custom fields

## Results

### Step 1: Definition of analytical and operational objective

Following step 1, we defined the Analytical Target Profile for the chosen use case on galactosylation adaptive control (Table 4). The Analytical Quality by Design (AQbD) framework represents a science and risk-based approach for developing robust analytical methods that comply with regulatory requirements in the pharmaceutical industry [29]. The elements of AQbD are outlined in the ICH14 as an enhanced approach for analytical procedure development [30, 31]. We adopted the minimal approach following the ICH14 recommendations to develop a robust analytical method that is fit for the intended purpose of monitoring the major glycan species to enable adaptive process control. The approach began with the identification of critical product attributes that necessitated testing, specifically focusing on glycosylation as a critical quality attribute (CQA). Of note, the reportable range is only an example. Based on the criticality it must be adapted for a therapeutic product.

**Table 4:** Analytical Target Profile – adaptive control of galactosylation

Category	Description
Intended Purpose	Real-time process monitoring and adaptive control of galactosylation levels in monoclonal antibody production during upstream bioreactor run
CQA	Glycosylation—specifically galactosylation (G0, G1, G2) affecting Fc-mediated effector functions
Performance Characteristics	Method performance for specificity, linearity, accuracy, precision and limit of quantitation
Reportable Range	Galactosylated Fc glycans present within a relative abundance of more than 5%
Decision Time	8 h (sampling to results with adaptive control response)
Technology	Established as at-line, with minimal sample preparation for monitoring the major glycan species

**Fig. 2:**
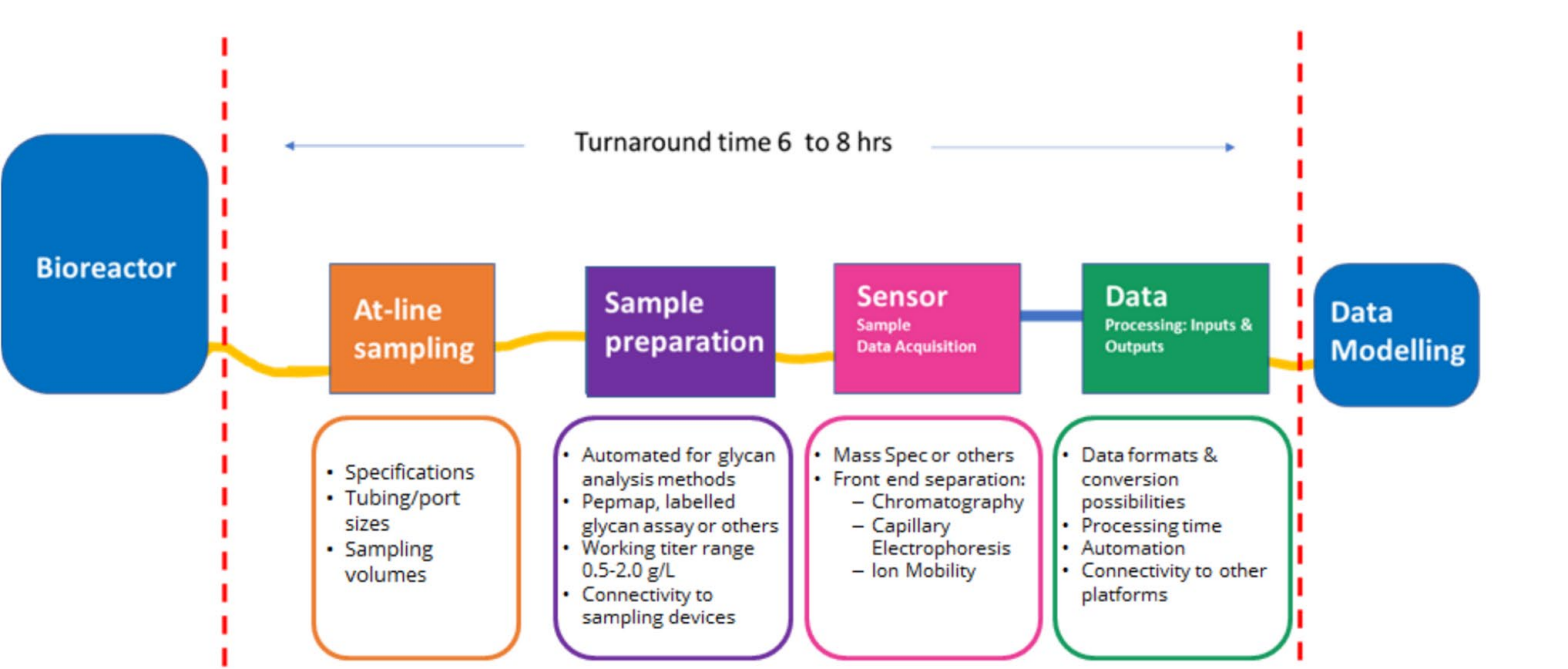
User requirements for glycan monitoring in upstream unit operation for model-based control

The user requirement for the at-line glycan measurement within the upstream unit operation was defined from the inputs collected from participants (industry, academic and suppliers) within the National Institute for Innovation in Manufacturing Biopharmaceuticals (NIIMBL) Big data program (Fig. 2). The user requirement elaborated the needs from at-line sampling, glycan sample preparation, sensor data acquisition and data processing for monitoring the glycan distribution during the bioreactor run. The glycan analysis frequency was set at 8-h intervals, as meaningful changes in glycan profiles require 6 to 12 h to manifest following perturbations in pH, temperature, nutrients, and process parameters. Analytical Target Profile (ATP) was defined within the scope of adaptive control of galactosylation, requiring the measurements of the major galactosylated glycans consisting of either zero (G0), one (G1) or two (G2) terminal galactose moieties. The ATP captures the measurement needs, description of the intended purpose and the performance characteristics of the analytical method (Table 4).

### Step 2: Identification of candidate analytical technologies

**Fig. 3:**
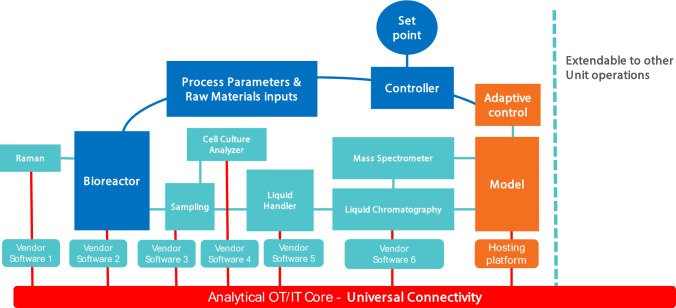
Analytical technologies and architecture for adaptive control of glycosylation

**Fig. 4:**
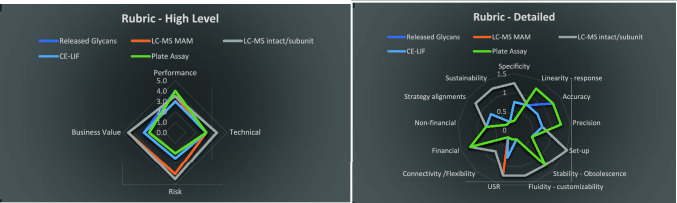
Outcome of rubric results for glycan analytics technology assessment

Following step 2 of technology assessment, the current analytical technologies for glycosylation analysis were explored. The choice of analytical method depends on the business objectives, analyte identification capabilities and practical considerations for at-line sample preparation and data availability in real-time. The comparative analysis of various glycan analysis techniques highlights their distinct capabilities and limitations (Table 5). The intact glycan analysis method, which identifies approximately 80% of glycans, offers good composition analysis but lacks isomer differentiation and branching confirmation [32–34]. Subunit or IdeS analysis improves the intact method by identifying around 85% of glycans, offering better relative quantification and similar limitations regarding isomer detection. Glycopeptide analysis further refines these capabilities, identifying up to 95% of glycans with a good relative quantification. However, it still lacks isomer differentiation. Released and labeled glycans analysis provides the glycan identification rate of 99.9%, with good relative quantification. With the use of advanced mass spectrometry techniques, it is possible to confirm glycan structural branching. However, this method requires multiple sample preparation steps that are performed using commercial kits, which are available for short (2 to 4 h) and long (overnight deglycosylation, labelling and clean-up: 1 to 2 days) duration formats. The multiple steps for sample preparation, and the necessity to store reagents at-line, while maintaining stability poses a limitation for its use as a PAT for real-time process monitoring. Based on the feedback provided by the industry, academic and institution participants in the NIIMBL Big Data program- interoperability stream, five commonly used glycan analytics were identified as most comprehensive, efficient, or robust methods currently available for glycan measurements (Figs. 3, 4).

**Table 5:** Capabilities of Various Glycan Analytical Technologies

Glycan Analytical Technologies	% Glycans Identified (estimated)	Composition	Isomers	Relative Quantity	Online Capability	Other CQAs	Time
Intact molecule	+ + +	+ + + +	-	+ +	+ + + +	+ +	+ + + +
Subunit	+ + +	+ + + +	-	+ + +	+ + + +	+ +	+ + +
Glycopeptide analysis	+ + + +	+ + + +	-	+ + + +	+ + +	+ + + +	+
Released glycans (non-labeled)	+ + + +	+ + + +	+	+ + + +	+ + +	-	+ +
Released and labeled	+ + + +	+ + + +	+ + +	+ + + +	+ +	-	+
Capillary Electrophoresis with laser induced fluorescence (CE-LIF)	+ + +	+ + +	+ + + +	+ + +	-	-	+
Plate Assay	+	+	+	+ +	-	-	+ +

( +) indicates capabilities that are achieved with each glycan analytical technology. Number of ( +) corresponds with strength of each capability, ( +) Basic capability, (+ +) Moderate capability, (+ + +) High capability and (+ +  + +) Comprehensive capability. (–) indicates the capability is not achievable with this method

### Step 3: Systematic evaluation of analytical technologies

Following step 3 of the technology assessment, the chosen glycan analytics were assessed by using the technology assessment rubric (Table 6). The heat map employs a color gradient, wherein green indicates high scores representing desirable characteristics. While red represents low scores with not fully aligned characteristics, and intermediate colors (yellow and orange) indicate moderately aligned desired characteristics. In-depth evaluation revealed that released glycan analysis involves enzymatic cleavage of glycans from glycoproteins using enzymes such as PNGase F, followed by fluorescent labeling with dyes like 2-aminobenzamide (2-AB) or 2-aminoanthranilic acid (2-AA). The labeled glycans are analyzed using ultra-performance liquid chromatography (UPLC) coupled with fluorescence detection (FLD) and/or mass spectrometry (MS) to determine structure, composition, and abundance. This method is considered the “gold standard” for glycan analysis due to its excellent linearity, accuracy, and precision, making it widely accepted by regulatory agencies for quality control testing. However, the complex multi-step sample processing limits its use for at-line process monitoring in manufacturing environments. Liquid Chromatography-Mass Spectrometry (LC–MS) Multi-Attribute Method (MAM) enables simultaneous monitoring of multiple critical quality attributes including site-specific glycosylation patterns, amino acid sequence variants, and post-translational modifications such as deamidation and oxidation. The method involves enzymatic digestion of glycoproteins into peptides and glycopeptides, followed by LC separation and MS detection. LC–MS MAM provides high specificity with site-specific glycosylation information and relative quantitation of glycan species. However, the multi-step sample preparation, extensive processing steps, and long analysis time, limit its use for real-time data generation. Additionally, complex mass spectrometric data requires dedicated software and highly skilled personnel for processing and interpretation of results. LC–MS analysis of intact mAbs or partially digested mAbs as subunits, provides sufficient information about the overall glycosylation profile of the therapeutic mAb [32, 34]. This method has simpler sample preparation and involves chromatographic separation to provide relative glycan distribution. The analysis may also involve a mass spectrometer following the chromatographic separation to determine the molecular weight and quantitation by MS [35]. Capillary gel electrophoresis with laser-induced fluorescence detection (CGE-LIF) leverages fluorescent labeling and electrophoretic separation for high-resolution glycan profiling [36–38]. This method provides high sensitivity, relative quantitation, and can differentiate closely related glycan structures within a short run time. However, the method relies on fluorescent labeling and offline handling, which limits automation and online integration capabilities. Plate-based glycan screening methods, including lectin microarrays and microtiter-plate assays, are able to analyze comparative “glycan fingerprints” across multiple samples with minimal turnaround time [39, 40]. These platforms enable high-throughput analysis and targeted assessment of specific glycan features (sialylation, high mannose, galactosylation) but cannot identify individual glycan species or provide species level quantification. The technology assessment for at-line galactosylation control in mAb manufacturing concluded that LC–MS subunit (middle-up) analysis provides the most favorable balance of analytical information and at-line operational readiness. The method was deemed fit-for-purpose to control the galactosylation, as it requires minimal sample preparation and provides the glycan distribution of major glycoforms within a short time to meet the control objectives.

**Table 6:**
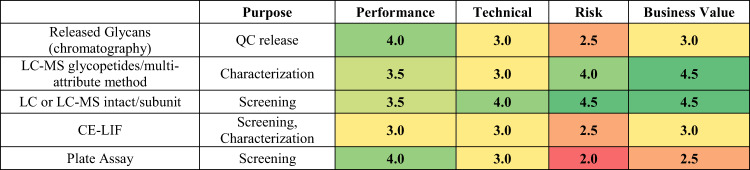
Outcome of overall Technology Assessment for glycan analytics

### Building the interoperable analytical system for glycan adaptive control

**Fig. 5:**
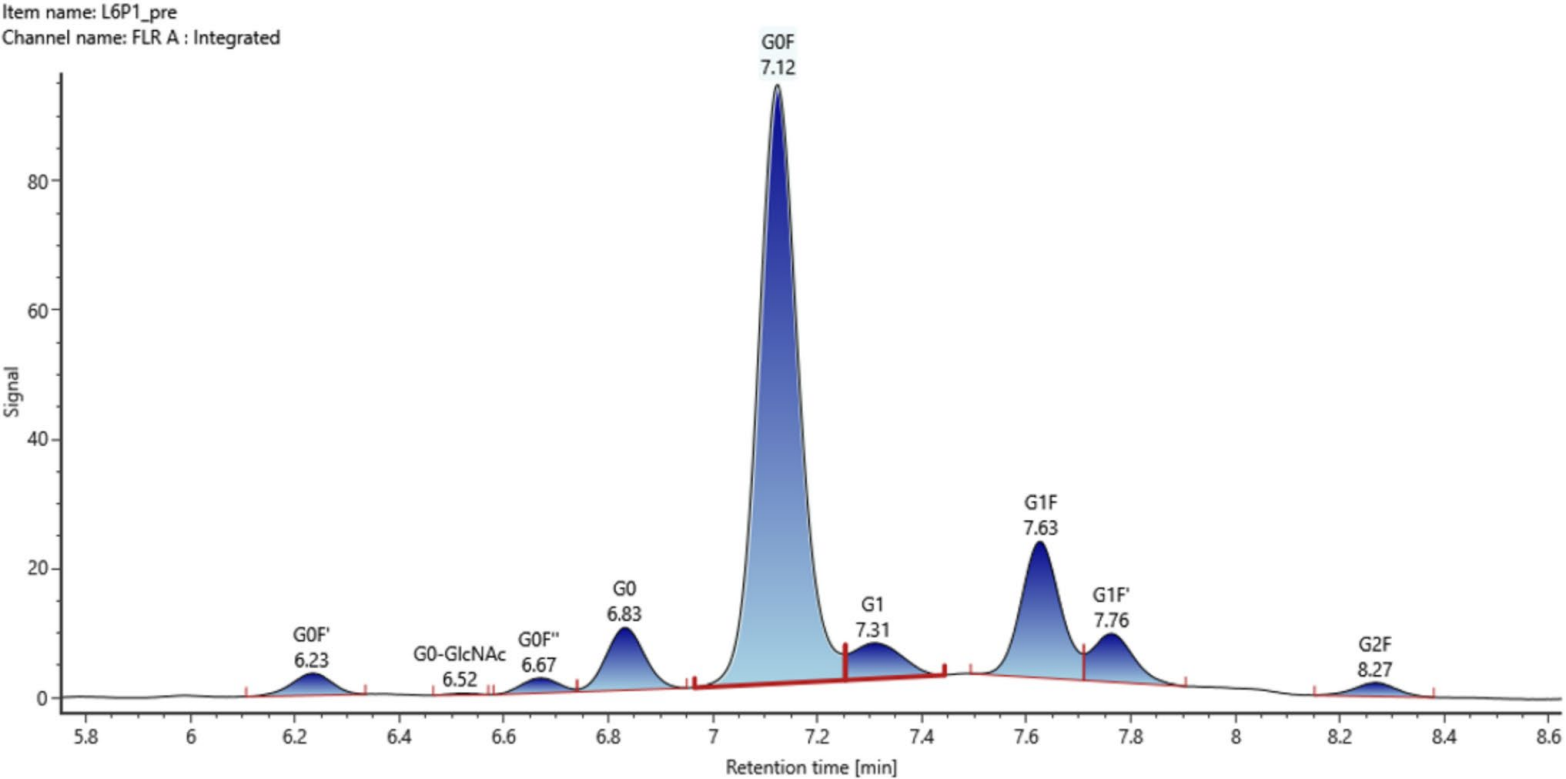
Identity of the LC-subunit FLR chromatographic peaks as assigned by mass spectrometry

Method development work was focused on developing a rapid subunit-level glycan analysis method suitable for at-line measurements on the BTEC Testbed for enabling real-time process control. The method aims to monitor major glycan species (e.g., G0F, G1F, G2F) during bioreactor operations, targeting a turnaround time of ≤ 8 h from sample collection, data generation, and providing a decision for adaptive control response. Sub-unit glycan method utilized IdeS, which is a cysteine protease that specifically cleaves human immunoglobulin G (IgG) antibodies at the hinge region, resulting in the generation of two distinct fragments as Fc (fragment crystallizable) and the F(ab’)2 (fragment antigen-binding). Upon further reduction of disulfide bonds, three components as Fd (fragment antigen-binding), light chain fragment, and scFc (single-chain fragment crystallizable) containing one glycan site are released [41]. HILIC chromatography allowed the separation of the light chain, Fd and multiple peaks corresponding to scFc carrying different N-glycan species. Initial method development utilized Waters lyophilized monoclonal antibody subunit standard to evaluate HILIC chromatographic separation performance. The identity of the peaks in the HILIC chromatography profile was assigned from the mass spectrometric data (Fig. 5, and details in supplementary Table 2). Fluorescence (FLR) detector chromatograms demonstrated baseline or near-baseline resolution of subunit fragments as light chain, Fd, and scFc identified as multiple peaks corresponding to different glycoforms. Quantification was performed using the FLR peak area for the predominant glycoform species confirmed by MS at each retention time. The assignments from MS data were used to build a glycan retention time library for FLR-based quantitation. The main species were observed as G0, G0-GlcNAc, G0F, G1, G1F, G2F, and close to baseline levels G2F + 2SA, and G2F + SA (due to their inherent lower levels in the sample). Our observations were consistent with the literature [35], confirming sufficient chromatographic peak separation resolution, and FLR based glycoform-level quantitation. Mass spectrometric (MS) signals are generally more variable than fluorescence (FLR) signals due to ionization efficiency, matrix effects, and instrument drift, leading to variability between runs and samples. Comparatively, the FLR signal provides good sensitivity and robustness for the major glycan species by detecting up to 0.1% relatively abundant glycan species.

The method developed from BTEC Testbed was assessed for the analytical performance at the NIIMBL Testbed at University of Delaware. This required the adaptation of the developed protocol to the different instrumentation. The subunit analytical method was assessed for performance using the recommendations from ICH Q14 guidelines for a minimal approach [30]. The mAb-free sample showed no peaks, while the mAb-containing sample displayed the expected peak profile, confirming method specificity. Linearity was assessed at five levels (50%, 75%, 100%, 125% and 150%) for each major glycan species (G0, G0-GlcNAc, G0F, G1, G1F, G2F), within a relative distribution range of 0.9% to 45%. The linearity showed good statistical significance R^2^ = 0.96—0.99 across all major glycan species (Supplementary Fig. 1). The predominant glycoforms G0F (43.4% relative distribution, R^2^ = 0.9935) and G1F (24.2% relative distribution, R^2^ = 0.9898) exhibited excellent linearity, while minor species G0-GlcNAc (2.3% relative distribution, R^2^ = 0.9608) and G2F (0.9% relative distribution, R^2^ = 0.965) showed acceptable linearity with slightly reduced correlation. This was due to their lower abundance and proximity to detection limits. The linearity results demonstrated consistent analytical response across the concentration range, essential for accommodating variable mAb titers throughout bioreactor operation. Accuracy evaluation was performed to confirm the closeness of agreement between measured values and theoretical reference values through percent recovery calculations on each glycan species, with overall values between 82–119%. The relative distribution of predominant glycoforms G0F (43.4%) and G1F (24.2%) exhibited excellent accuracy with recovery ranges of 91–103% and 87–107%, respectively (Supplementary Table 3). Precision was determined through relative standard deviation measurements to confirm repeatable and consistent results across multiple analysis. Precision evaluation showed variable performance across glycan species, with relative standard deviations (%RSD) ranging from 4 to 42%. The major glycoforms G0F, G1F, and G1 demonstrated acceptable precision with %RSD values of 5%, 4%, and 8%, respectively, indicating reliable quantification for these abundant species. However, the minor glycoform G2F exhibited high precision variability (%RSD = 42%), attributed to low species abundance (Supplementary Table 3). The sub-unit method demonstrated acceptable linearity, accuracy and precision for the purpose of monitoring major glycoforms representing > 95% of the total glycan profile, while minor species quantification may require additional method development or the use of high sensitivity methods.

**Fig. 6:**
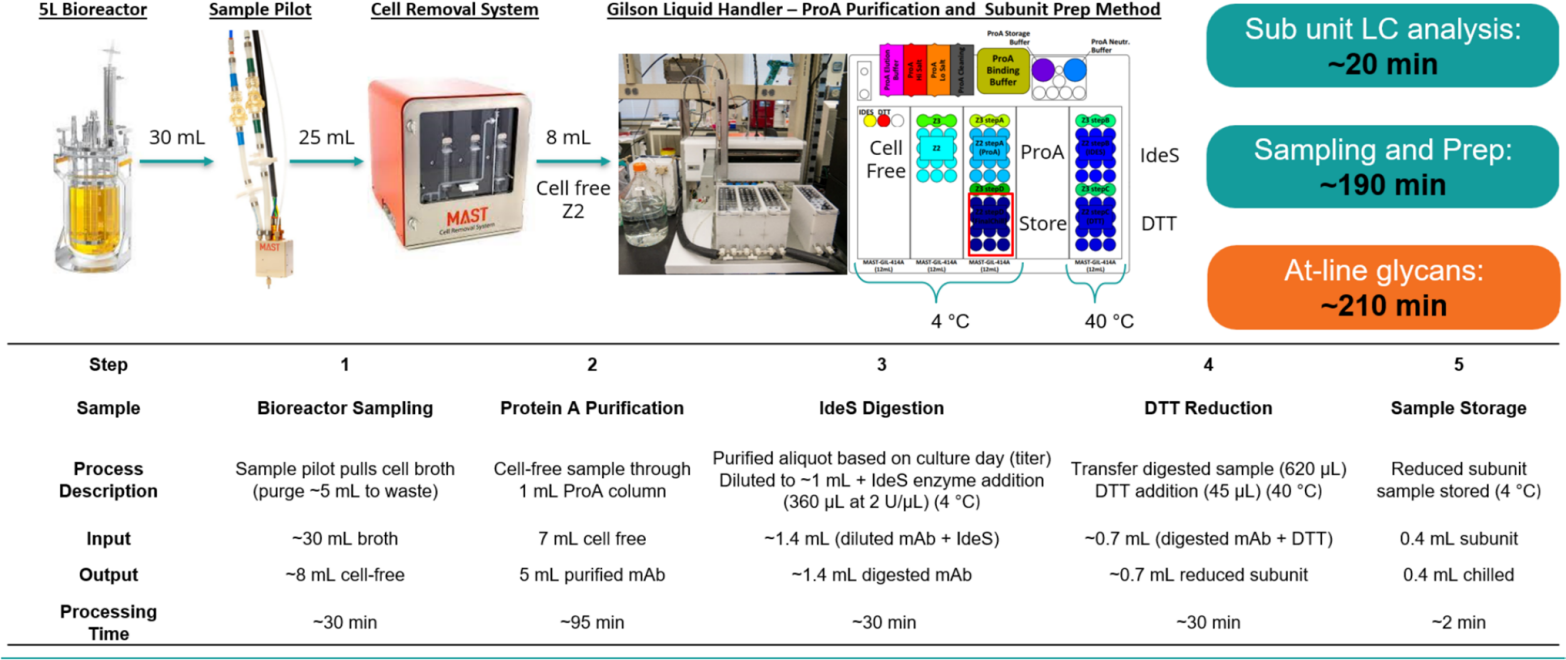
At-line adaptation of the sub-unit glycan method at BTEC Testbed

Real-time glycosylation monitoring has emerged as a critical enabler to manage the dynamic glycosylation changes during the bioreactor run. The offline LC sub-unit method was adapted for the at-line set-up as described in the materials and methodology section. The adapted at-line LC sub-unit method met the criteria for the analytical target profile (Table 4) and user requirements (Fig. 2). End-to-end execution from the bioreactor sampling, sample preparation and analysis by LC chromatography was completed in 210 min (Fig. 6). Previous studies have used similar approach for at-line analytics with a low-volume and high-throughput approach for at-line product quality analysis [42]. An automated system comprising of micro sequential injection (μSI) coupled with UPLC was used for monitoring real-time antibody glycan profiles to study the manganese (Mn)-induced glycosylation changes [43].

Mechanistic mathematical models have shown to predict glycosylation profiles by accounting for enzyme kinetics, Golgi maturation, and nucleotide sugar donor transport [44–46]. Several studies have systematically evaluated the media design and supplementation strategies for glycosylation modulation [46–49] for mAbs, as well as demonstrated biological activity differences on complex mAb based fusion molecules having several glycosylation sites [50]. Feed substrates with varying concentrations of uridine, manganese chloride, and galactose have shown to enable glycosylation modulation with the rapid transition of G0F to G1F [51, 52]. Among the various supplements, galactose and glucosamine have emerged as key modulators of galactosylation. Galactose supplementation increased galactosylation up to 21% in CHO cell cultures, with no adverse effect on cell growth or titer [53]. In perfusion cultures, galactose and manganese supplementation significantly increased the proportion of complex galactosylated glycans, and the changes were maintained at steady state for over 20 days [46]. Galactose and manganese additions affected the galactosylated glycans in the mAb, reaching 70% with alternate day feeding for a fed-batch culture, and 55% for perfusion culture, compared with the control 25% [54]. Glucosamine addition at 12.5 mM as a feed, significantly reduced galactosylation at the species levels for G1, G1F, and G1F’ by 85%, 50% and 62% respectively [49]. Within the NIIMBL Big Data program, a perturbant impact assessment study was conducted on galactosylation (data not included). Design of experiments were conducted to evaluate the impact of process perturbants on galactosylated cNISTmAb-BTEC production under controlled bioprocess conditions at small scale-Ambr® 250. Thirty-six experimental conditions were investigated using the small-scale bioreactors, and time-course glycan profiling was performed for all bioreactor runs (over 200 bioreactor samples) and analysed with the sub-unit glycan method. Statistical analysis of the resulting glycan profiles identified specific process perturbants that demonstrated significant impact on galactosylation patterns, establishing their suitability for implementation in galactosylation adaptive control. The effects of perturbants in changing the galactosylation profile was captured by the sub-unit glycan method, which was able to differentiate and quantify the major species to facilitate the generation of a high-quality data set for fine tuning the glycan adaptive control model (data not included).

The model-based glycosylation adaptive control requires the integration of real-time analytical monitoring and predictive process control to perform dynamic optimization of N-glycosylation profiles in therapeutic monoclonal antibody production. The adaptive control of glycosylation in mAbs (IgG1), particularly produced in Chinese Hamster Ovary (CHO) cells, can be effectively controlled by focusing on a minimal set of glycan species as captured by the LC sub-unit glycan method. Additionally, the abundant glycans (G0F + G1F + G2F) constitute a significant proportion of the overall glycan distribution for therapeutic mAbs (IgG1) produced from either of the three cell lines (CHO, NS0, and Sp2/0) [52]. Within the NIIMBL Big Data program, a hybrid model for the glycosylation distribution prediction and the subsequent control response for the addition of supplements was developed using both mechanistic and data driven approaches, and was fine-tuned with the data generated by the LC based sub-unit glycan method from the design of experiment studies (data under proprietary review and will be published as a separate technical article). The description provided here is scoped to convey the model’s structural role within the digital twin architecture, its input–output specification, and the data generation strategy underpinning it. The model required multiple input parameters including glycan distribution from the LC subunit method for the major glycan species (G0, G0-GlcNAc, G0F, G1, G1F, G2F), process parameters such as input flow rates for base addition (pH control), antifoam, nutrient feed supplementation, and output flow rates for sampling the media composition for key metabolites (glucose, glutamine, glutamate, lactate, and ammonia) critical to cellular metabolism and glycosylation pathways; and modulators (galactose, asparagine, aspartate, and uridine) that directly influence nucleotide sugar metabolism and glycan biosynthesis. Further model inputs involved cell culture parameters as viable cell density, total cell count, and viability for understanding the culture’s physiological state for optimized cell growth and productivity. The model output generated targeted control responses through optimized feed pump flow setpoints for precise volumetric additions, enabling closed-loop control of glycosylation profiles through modulator supplementation. This interoperable system enables the convergence of multiple data sources to facilitate the establishment of a closed-loop control system that can dynamically adjust process parameters based on real-time glycosylation distribution. We performed two 5 L bioreactor runs to demonstrate model-based predictive control (MPC) of galactosylation. One run was operated under standard conditions with a glucosamine perturbation on day 5 and no control response. The second run included the same day 5 glucosamine perturbation but implemented MPC from day 6 onward, targeting 27% galactosylation (± 3% deadband) by adjusting galactose addition to maintain the setpoint. The at-line LC subunit method was able to detect the major galactosylated glycan species (Supplementary Table 4). The assessment was performed by grouping glycans with terminal, exposed galactose as galactosylated, while all other glycans were grouped and summed as non-galactosylated. The fucosylated group was calculated from the sum of all the fucosylated glycan species. The galactosylation index was calculated for standardized assessment on comparability using the methodology provided by [28]. In the perturbated bioreactor, where no adaptive interventions were implemented, a progressive decrease in galactosylated species was observed from 29.3% on Day 6 to 10.5% on Day 14, representing a substantial 14.6% change from the maximum galactosylation on day 6 (Table 7). The galactosylation index [28], indicated significant differences for Index G0, Index G1 and Index G2 between day 6 (0.71, 0.27, and 0.02, respectively) and day 14 (0.90, 0.11, and < 0.01, respectively). The perturbated bioreactor with model-based prediction for supplementation of galactose was able to maintain the set level of galactosylation. The glycan monitoring from day 6 to day 14, exhibited a range of 15.4% to 28.2% for the galactosylated groups, but with the final day 14 value at 24.6% representing only a 2.4% difference from the 27% setpoint (within the ± 3% deadband), compared to the 14.6% difference observed in the perturbated bioreactor without feedback control. The comparative analysis of glycosylation profiles between perturbated and perturbated with adaptive control bioreactor runs demonstrated the successful application of model based predictive control in maintaining the targeted galactosylation levels through the supplementation with galactose. The results established the feasibility of real-time practical implementation of model-based predictive control of glycosylation to achieve a reduction in galactosylation variability, and to maintain a consistent desired galactosylation distribution.

**Table 7:** Galactosylation & fucosylation assessment in perturbated and adaptive control bioreactor run over time

Run	Culture Day	Fucosylated	Non-Galactosylated (G0F, G0, G0-GlcNAc, G2F + 2SA)	Galactosylated (G1, G1F, G2F, G2F + SA)	Change in Galactosylation from Day 6	Change in Fucosylation from Day 6	Index G0	Index G1	Index G2
Perturbated	Day 5	95.9%	74.9%	25.1%	< 0.1%	< 0.1%	74.9%	25.1%	< 0.1%
Perturbated	Day 6	94.4%	70.8%	29.3%	4.1%	-1.5%	70.8%	27.4%	1.9%
Perturbated	*Day 7	–	–	–	–	–	–	–	–
Perturbated	Day 8	88.4%	80.5%	19.5%	-5.6%	-7.4%	80.5%	18.7%	0.8%
Perturbated	Day 9	88.0%	78.1%	21.9%	-3.2%	-7.8%	78.1%	21.3%	0.6%
Perturbated	Day 10	87.6%	77.0%	23.0%	-2.1%	-8.2%	77.0%	22.6%	0.5%
Perturbated	Day 11	85.5%	77.5%	22.5%	-2.6%	-10.4%	77.5%	22.5%	< 0.1%
Perturbated	Day 12	79.3%	80.5%	19.5%	-5.7%	-16.6%	80.5%	19.5%	< 0.1%
Perturbated	Day 13	79.7%	83.4%	16.6%	-8.5%	-16.2%	83.4%	16.6%	< 0.1%
Perturbated	Day 14	85.7%	89.5%	10.5%	-14.6%	-10.1%	89.5%	10.5%	< 0.1%
Adaptive Control	Day 5	93.7%	76.8%	23.2%	< 0.1%	< 0.1%	76.8%	21.8%	1.5%
Adaptive Control	Day 6	92.2%	71.8%	28.2%	5.0%	-1.6%	71.3%	26.8%	1.9%
Adaptive Control	Day 7	93.9%	77.8%	22.2%	-1.1%	0.1%	77.8%	21.1%	1.0%
Adaptive Control	Day 8	88.9%	80.0%	20.0%	-3.3%	-4.8%	80.0%	19.2%	0.8%
Adaptive Control	Day 9	81.4%	84.6%	15.4%	-7.8%	-12.4%	84.6%	15.4%	< 0.1%
Adaptive Control	Day 10	81.5%	80.8%	19.2%	-4.1%	-12.3%	80.8%	18.4%	0.8%
Adaptive Control	Day 11	81.7%	78.4%	21.6%	-1.7%	-12.0%	78.4%	21.6%	< 0.1%
Adaptive Control	Day 12	77.4%	79.2%	20.8%	-2.4%	-16.4%	79.2%	20.8%	< 0.1%
Adaptive Control	Day 13	75.7%	75.6%	24.4%	1.2%	-18.0%	75.6%	23.4%	1.0%
Adaptive Control	Day 14	74.6%	75.4%	24.6%	1.4%	-19.2%	75.4%	24.6%	< 0.1%

^*^Day 7 sample for the perturbated run is missing due to an error on the cell removal system resulting in no sample being pulled from the bioreactor at that time

## Discussion

The at-line subunit-based glycan analysis approach was systematically selected through a comprehensive technology assessment rubric, specifically designed to identify analytical methodologies suitable for big data applications. The technology assessment rubric employed a multi-criteria evaluation matrix encompassing performance metrics (specificity, linearity, accuracy, precision), technical readiness levels, risk assessment parameters, and business value considerations that are important drivers for decision making in the organizations. The rubric’s importance for decision-making extends beyond glycan CQA to a broader landscape of CQAs in biopharmaceutical manufacturing. This multi-criteria assessment can be applied to evaluate real-time analytical technologies for purity, fragments, impurities, aggregation, charge variants, host cell protein, residual DNA etc., to develop digital twin applications for a mAbs product. The technology assessment rubric establishes a standardized methodology for evaluating the process analytical technologies to enable systematic comparison. The framework’s applicability can be extended to other real-time CQA analysis such as protein titer through spectroscopic methods, continuous monitoring of product-related impurities via at-line chromatographic systems, and detection of process-related impurities through integrated sensor platforms. Currently, we have evaluated mAbs based CQAs, and the methodology can be generalized to any therapeutic product type.

Subunit-based glycan analysis approach was demonstrated to facilitate the model-based adaptive control of glycosylation in mAbs biomanufacturing. The method delivers a 210-min analytical turnaround time, encompassing automated sampling, sample preparation, chromatographic analysis, and data processing as an analytical workflow. We provide the feasibility for the real-time glycosylation monitoring and modulation, to achieve the target desired glycosylation profile with consistent product quality. A comprehensive survey of Biologic License Application (BLA) documents focusing on the N-glycan profiles of therapeutic monoclonal antibodies (mAbs) IgG1 produced in Chinese Hamster Ovary (CHO), revealed ten most prevalent glycan species as G0F, G1F, G2F, G0, M5, G0F-GlcNAc, G1, M6, G2F + NANA, G1F-GlcNAc [52], and additional prevalent glycan species were reported for the mouse myeloma cell lines (NS0 and Sp2/0). Interestingly, the top three glycans (G0F + G1F + G2F) across the three cell lines (CHO, NS0, and Sp2/0) showed the average narrow range of 82–90%, and a 65–99% of wide distribution range [52]. The sub-unit glycan method was able to cover ten most prevalent glycan species, as observed in our studies and as reported in the literature [35]. Notably, authors have provided a comparison between the 2-AB released glycan method and the subunit-based glycan analysis on Adalimumab, and showed a good analytical concordance of subunit-based glycan analysis method utilizing fluorescence detection (FLD) [35]. While the absolute quantitation may differ, the relative abundance trends and detectability of major glycan species were consistent between the two techniques. This enables the subunit LC glycan method as a rapid, fit‑for‑purpose process‑monitoring method, capable to indicate the similar trends, as that of the reference released‑glycan method. It should be noted that dedicated comparability studies are needed to establish the analytical equivalence between the sub-unit LC glycan method and the 2-AB based released glycan reference method for using it in a regulated environment. In our work, high mannose species were minimally present in the bioreactor samples, and the sub-unit LC method was not specifically optimized for resolving the mannosylated glycans. This was considered acceptable as our focus was on the galactosylated glycans for galactosylation control as per the analytical target profile. Future work should focus on optimizing the method for the detection of mannosylated species, particularly if high mannose is considered as a critical quality attribute for the therapeutic mAb product. Additionally, there may be other specific cases where either the method or the selection rubric needs to be revisited, e.g., control of low-level a-fucosylation (i.e. 1–4% range) or sialylation. The analytical performance characteristics of sub-unit LC glycan method showed acceptable linearity (R^2^ = 0.96–0.99), accuracy (82–119% recovery), and precision (%RSD = 4–42%, depending on glycoform abundance) for the predominant glycans. Preliminary results from the bioreactor runs demonstrated that the subunit glycan method successfully resolved and quantified major galactosylated glycoform species, enabling model-based control of galactosylation within the ± 3% deadband of the 27% target setpoint, without impacting fucosylation. Importantly, our work represents a feasibility demonstration of PAT for process control. Further work is required to cover all input variables as part of the system to enable full establishment in commercial biomanufacturing in alignment with regulatory expectations.

The findings from the use case of glycan adaptive control exemplify the complexity of establishing the digital twins in regulated biomanufacturing environments. Model based control systems require seamless integration of numerous analytical platforms, wherein each generates diverse data in various formats and communicates through different protocols. Our glycan adaptive control use case ecosystem included bioreactor, pumps, inline and at-line sensors (pH, dissolved oxygen, cell density, and metabolite concentrations), autosamplers, and analytical systems. These devices transmit data either as analog signals (continuously), serial connections (one bit at a time), discrete digital outputs (on/off as binary information), OPC DA/UA protocols (structured, multiple types), or file-based formats such as.CSV (processed data exports). This heterogeneity in data formats and communication protocols presents significant challenges for system validation and lifecycle management of a model-based control system. Validation of such multi-platform system is inherently complex, requiring verification of individual device accuracy and reliability, while ensuring data integrity and traceability across the entire system, from raw signal acquisition to actionable process control outputs. Any change in data format, software version, or communication protocol would disrupt the established data pipelines, necessitating complete system revalidation. With the evolution of new technologies and platforms, there is a high risk of incompatibility and data inconsistency. Newer instruments, sensors, and software solutions often employ unique or different data formats, proprietary communication protocols, or novel integration standards that may not be readily compatible with existing infrastructure and legacy systems. The BTEC Testbed deployed for a glycan adaptive control use case highlights the operational complexity and limitations to implementing model-based predictive process control. Four principal gaps were identified: (i) physical and software integration: between the liquid handler and the LC–MS instrument; (ii) data exchange silos: between the LC–MS and process data ecosystem; (iii) model input/output data flows: manual Excel-based data handling, and iv) connectivity and communication: model outputs to the control system. The set-up required manual operations to load the samples on LC–MS from liquid handler, due to the absence of universal connectors and orchestration platform. The LC–MS data processing was also performed manually, due to incompatibilities between instrument-specific APIs and universal middleware at the BTEC Testbed. Furthermore, the analytical data from sensors were manually consolidated into an Excel file and predicted control actions were manually entered by an operator to execute the prescribed control response. This led to challenges in establishing seamless data exchange, resulting in information silos and loss of interoperability between critical system components. Data inconsistency arises, as different devices interpret, store, or transmit the same information or data in different ways. These discrepancies impact standardizations, data integrity, and the reliable functioning of model-driven process control strategies.

Our findings provide a guiding path to apply the Interoperability “Triangle” connecting the digital twin dimensions with the interoperability levels (Table 8). The consistent data generation across different workflows (Data, Physical Entities) and their exchange in standard formats (L2) with semantic meaning (L3) are foundational for ensuring that information remains interpretable and actionable across platforms. Coordinated execution by multiple devices and systems (Physical Entities, Connection, Services) is enabled by basic connectivity (L1), standardized data (L2), and meaningful exchange (L3), allowing for synchronized process operations. Seamless integration and synchronized operation require not only connectivity and data standards but also context-aware data exchange (L4) that supports real-time, adaptive decision-making (L5). The ability to aggregate multiple data sources for real-time control (Data, Services, Connection) and enabling the adaptation across different instruments and manufacturing sites (Services, Connection, Physical Entities), ensures that the digital twins are robust, scalable, and aligned to evolving business and regulatory requirements (L6). Meaningful data exchange and contextual bridging (Data, Virtual Entities, Services) ensure that digital models and analytics retain their relevance and accuracy across scales and lifecycle. A practical instantization of Glycan Adaptive Control use case is provided in Table 9.

**Table 8:** Interoperability Levels and Digital Twin Dimensions in Glycan Adaptive Control Systems

Glycan Adaptive Control	Digital Twin Dimension(s)	Interoperability Level(s)	Description
Consistent data generation across different workflows	Data, Physical Entities	Standard Data (L2), Meaningful exchange (L3)	Meaningful Information is exchanged across the different systems in a consistent and standard format
Coordinated execution by multiple diverse combinations of devices/systems	Physical Entities, Connection, Services	Connectivity (L1), Standard Data (L2), Meaningful exchange (L3)	Basic connectivity, standardized formats, and semantic understanding enable coordination
Seamless integration and synchronized operation of devices/systems	Connection, Services, Physical Entities	Connectivity (L1), Standard Data (L2), Meaningful exchange (L3), Contextual relevance and actions (L4)	Connectivity, data standards, meaningful and contextually relevant data exchange allow seamless and synchronized operation
Multiple data sources for real-time control	Data, Services, Connection	Contextual relevance and actions (L4), Adapting to real-time data (L5)	Meaningful data exchange enable real-time, contextual data integration for control
Method adapted with different instrumentation & sites	Services, Connection, Physical Entities	Contextual relevance and actions (L4), Adapting to real-time data (L5), Aligning to business objectives (L6)	Interoperability enables adaptation across sites and instruments, aligning with business objectives
Meaningful data exchange and maintaining contextual relevance	Data, Virtual Entities, Services	Meaningful exchange (L3), Contextual relevance and actions (L4), Adapting to real-time data (L5)	Context-aware, dynamic data bridging ensures models and analytics remain relevant across scales

**Table 9:** Instantization of Glycan Adaptive Control System

Interoperability Level	Glycan Adaptive Control	5-dimension framework digital twin
L1: Technical	Real-time sensor data exchange from bioreactor, autosampler, pumps, analytical systems	*Physical Systems:* Bioreactor, pumps, balances, flowmeters, FLEX2 Bioanalyzer, Cedex Bioanalyzer, LC–MS, MAST autosampler, Gilson liquid handler *Connections:* Analog, serial, OPC DA/UA, discrete, .csv data transfer
L2: Syntactic	Processed data into standardized formats (e.g., grouped glycan values in .csv, amino acid analysis, bioreactor sensors.)	*Data:* Standardized data structures for glycans, metabolite levels, amino acids, cell culture attributes, process parameters (pH, DO, etc.) *Connections:* Analog, serial, OPC DA/UA, discrete, .csv data
L3: Semantic	Meaningful data exchange to enable decisions (e.g., linking process parameters with glycan outcomes)	*Data:* Annotated and contextualized data e.g., time-stamped glycan profiles linked to feed rates and process conditions (DOE) *Virtual Entities:* Model for predicting glycan profile and control response *Services:* Data interpretation, analytics, and reporting engines
L4: Pragmatic	Process decisions and context-aware adaptive control (e.g., adjusting feeds based on glycan trends)	*Services:* Model-based control algorithms, decision support systems *Data:* Real-time data, glycans, metabolite levels, amino acids, cell culture attributes, process parameters (pH, DO, etc.) *Connections:* Automated feedback loops from analytics to process actuators (e.g., feed pumps)
L5: Dynamic	Monitoring and adaptive control in real time, responding to process variability	*Services:* Adaptive control strategies, predictive analytics *Virtual Entities:* Real-time digital twin continuous, model-based adjustments to maintain optimal glycosylation outcomes *Connections:* integration across the physical & virtual entities
L6: Organizational	Alignment to business objectives—quality, compliance and operational efficiency	*Services:* Quality management systems *Data:* Audit trails, compliance records, batch histories *Physical Entities:* Integration with enterprise systems (ERP, LIMS) and databases

The FDA draft guidance on AI use in regulated environments [55] recommends a risk-based credibility assessment framework encompassing: (1) definition of the question of interest and context of use, (2) model risk assessment, (3) development and execution of a credibility plan for AI-driven adaptive control, and (4) documentation of results and deviations. Implementation of our findings in a regulated manufacturing environment requires a comprehensive credibility establishment plan that holistically addresses the entire control system, including all input and output variables and their interdependencies. In the present use case, the model inputs were derived from multiple at-line sensing analytical systems as the FLEX2 Bioanalyzer for key metabolic and physicochemical parameters (pH, pO₂, pCO₂, glutamine, glutamate, glucose, lactate, ammonium, sodium, potassium, calcium, osmolality, and viable/total cell density), Cedex Bioanalyzer for the measurements of galactose, asparagine, aspartate, mAb titer, and the LC–MS platform for sub-unit glycans. The model outputs comprised actionable process variables, specifically feed pump flow rates, which were translated into precise control response (e.g., galactose, etc.) addition volumes. This multi-platform analytical architecture represents a highly complex system for regulatory validation. Implementation requires the fit for purpose assessment of each constituent analytical platform used to generate model input variables. The model credibility plan must include detailed documentation of the model architecture and development methodology. This encompasses explicit specifications of model inputs and outputs, the scientific and engineering rationale for the modeling approach, and a detailed elaboration of all the data used, with respect to its relevance and representativeness for the intended context of use (COU). Model training procedures must be meticulously described, with clear differentiation between training and test datasets, and with specific justifications. Model evaluation should be performed using independent test data that accurately reflects the COU, with scientifically rational performance metrics. The plan must address known model limitations, potential sources of bias, and the boundaries of model applicability. Representative datasets should be collected across multiple batches, products, and process conditions to demonstrate generalizability and robustness. Following FDA draft guidelines, subsequent steps involve executing the plan, documenting results and deviations, and determining COU adequacy [55]. This structured approach guides establishment of credibility, reliability, and regulatory acceptability for glycan adaptive control systems in biomanufacturing with a risk-based approach. Comprehensive documentation strategies, transparent reporting, and traceability of model development, validation, and deployment activities are needed to ensure system auditability, to meet regulatory expectations on commercial biomanufacturing operations.

## Conclusion

We have elucidated a merged digital‑twin and interoperability framework to advance digitalization in biomanufacturing for real-time process monitoring and model-based process control. This addresses a critical gap in biopharmaceutical manufacturing, where traditional PAT implementations often remain siloed and passive, limiting their potential for active integrated process optimization in real time. By mapping the five digital‑twin dimensions (Physical, Virtual, Data, Connection, Services) to six interoperability levels (Technical, Syntactic, Semantic, Pragmatic, Dynamic, Organizational) and aligning with ISO 23247, we provide an operational blueprint for establishing adaptive control in a regulated biomanufacturing environment. The resulting interoperability “triangle” provides a guiding framework that highlights how the Data and Connection dimensions are central to linking Physical and Virtual entities with value-generating Services, as demonstrated by MPC-enabled PAT that can respond dynamically to process variations in real time.

The integration of PAT to enable digital twin applications in biomanufacturing necessitates a systematic, purpose-oriented approach. The methodology begins with the definition of an Analytical Target Profile (ATP) that captures the intended purpose along with the critical decision window requirements essential for process control. Given the vast landscape of available process analytical technologies, a systematic technology assessment is required for assessing the capabilities and limitations of the technologies in relation to the intended purpose as per the analytical target profile. To assist in the selection of suitable analytical technologies we have defined a comprehensive technology assessment rubric that evaluates the technologies across analytical performance, technical readiness and operational setup requirements, risk factors, and business value indicators. This assessment drives the practical selection of analytical technologies for the identified analytical target profile to deliver the foreseen value.

With glycan adaptive control, we illustrate general guidelines for advancing digital twins in biomanufacturing. Our work elaborated the practical implementation of interoperability components across technical, syntactic, semantic, pragmatic, dynamic, and organizational levels, merged with the digital twin dimension framework. We elaborate the necessity of a system to achieve consistent data generation across diverse analytical workflows through standardized data formats and meaningful information exchange. This coordinated execution of multiple device combinations is enabled by connectivity, data standards, and semantic understanding for contextually relevant information flow. This facilitates value driven decision making in alignment with business objectives. The example case of MPC‑enabled glycan control can be extended to other critical quality attributes and provides a foundation for digital twin-based optimization of a bioprocess with controlled quality profile. We contribute towards theoretical advancement in digital twin conceptualization, and provide practical tools for development and implementation, to bridge the gap between Industry 4.0 concepts and their realization in highly regulated pharmaceutical manufacturing environments.

The adoption of digitalization facilitates the transformation of manufacturing processes into more intelligent operations, enabling real-time data collection, advanced analytics, and automated control systems [17, 56]. In the biopharmaceutical industry, the implementation of digital twins can enhance process understanding, optimize operations, and support decision-making through predictive simulations and real-time monitoring. Although the healthcare sector has been slower to adopt these technologies compared to other industries [57], there is a growing recognition of their potential to develop faster newer therapeutics and provide optimization for cost-effective production (17, 56, 58, 59). Particularly, the establishment of human oversight and risk-based approaches to AI governance is accelerating the maturation of advanced digital solutions. Beyond its technical feasibility, the presented glycan adaptive control use case highlights the tangible business and operational benefits of interoperable digital twin implementations. Maintaining galactosylation within a defined target range across the bioreactor run directly supports batch-to-batch comparability and provides a foundation for real-time release testing. Compared to traditional development approaches that rely on extensive experimentation, the adaptive control reduces the number of required experiments, minimizes manual data handling, and decreases the risk of human error associated with fragmented workflows. Automated, at‑line analytics coupled with predictive control reduce analytical cycle time and operator intervention while increasing data consistency and auditability. For biosimilar developers, the ability to dynamically control glycan profiles toward a predefined target during manufacturing represents a transformational approach to enable a greater assurance of product comparability, and increased flexibility in responding to process variability. More broadly, this work demonstrates how purpose‑driven integration of PAT, interoperability, and digital twin concepts can translate Industry 4.0 principles into measurable value within regulated biomanufacturing. Although, the path from testbed to GMP implementation requires extensive work in system validation, regulatory credibility establishment, and model lifecycle management. Our present work provides the operational blueprint and the demonstrated feasibility to justify the investment.

## Supplementary Information

Below is the link to the electronic supplementary material.

Supplementary file1 (DOCX 50 KB)

## Data Availability

This manuscript reports experimental bioprocess and analytical data (including glycan analytics and PAT/MPC testbed results), which were generated and analyzed as part of the study. Additional data are provided in the supplementary tables as well.
