# Supplementary material for: Operationalizing digital twins in biomanufacturing through interoperable process analytical technology
Source: Bioprocess Biosyst Eng. 2026 Jul 16;49(8):2061–83. doi: 10.1007/s00449-026-03369-9 (PMC13424265; doi:10.1007/s00449-026-03369-9)
Supplement: Supplementary file 1 — Supplementary file1 (DOCX 50 KB) [file 449_2026_3369_MOESM1_ESM.docx]

# **Annex/Supplementary information**

**Supplementary Table 1** At-line analytics and their turnaround frequency for model inputs.

| **At-line analytics** | **Model Input** | **Model Input Data** | **Automated or Manual** | **Turnaround Time** |
| --- | --- | --- | --- | --- |
| Glycan subunit analysis | Glycan Profile (% Relative Abundance) | G0F, G0, G1, G1F, G2F, G0GlcNAc, G2F+SA, G2F+2SA | Automated Sampling/ Manual Analysis | 210 min |
| Cedex Bioanalyzer | Media Composition (mmol/L) | Galactose, Asparagine, Aspartate | Automated Sampling/ Manual Analysis | 30 min |
|  | Product Titer (mg/L) | IgG |  |  |
| FLEX2 Bioanalyzer | Media Composition (mmol/L) | Glutamine, Glutamate, Glucose, Lactate, Ammonium | Automated | 10 min |
|  | Cell Density (cells/µL) | Total Cell Density, Viable Cell Density |  |  |
| DeltaV DCS | Inlet Flowrate (L/h) | Feed Pump Flowrates/Totalizers: Galactose, Glucose, Feed A, Feed B, Antifoam, Base | Automated | 1 sec |
| MAST Autosampler | Outlet Flowrate (L/h) | Calculated based on sampling schedule (fixed) | Manual  Calculation at start of experiment | N/A |
| DeltaV DCS | Bioreactor Volume (L) | Calculated based on initial weight | Manual  Calculation at start of experiment | N/A |

**Supplementary Table 2** LC-MS analysis for chromatography peak identification and assignments.

| **Component name** | **Observed RT (min)** | **Observed m/z** | **Response** | **Observed mass (Da)** | **Expected mass (Da)** | **Mass error (ppm)** |
| --- | --- | --- | --- | --- | --- | --- |
| oxFc Glycosylation G0F N (1) | 6.23 | 25231.1974 | 3.42E+03 | 2.52E+04 | 25231.9438 | -29.6 |
| redFc Glycosylation G0F N (1) | 6.23 | 25237.2178 | 1.20E+04 | 2.52E+04 | 25235.9756 | 49.2 |
| oxFc Glycosylation G0-GlcNAc N (1) | 6.52 | 24881.5831 | 4.86E+03 | 2.49E+04 | 24882.6101 | -41.3 |
| redFc Glycosylation G2F+SA N (1) | 6.52 | 25851.4617 | 4.06E+03 | 2.59E+04 | 25851.5114 | -1.9 |
| oxFc Glycosylation G0F N (1) | 6.67 | 25233.0498 | 1.13E+04 | 2.52E+04 | 25231.9438 | 43.8 |
| redFc Glycosylation G0F N (1) | 6.67 | 25235.8084 | 6.56E+03 | 2.52E+04 | 25235.9756 | -6.6 |
| oxFc +Lysine C-TERM (1), Glycosylation G0F N (1) | 6.67 | 25358.8899 | 3.71E+03 | 2.54E+04 | 25360.1161 | -48.4 |
| oxFc Glycosylation G0F-GlcNAc N (1) | 6.83 | 25028.6754 | 1.59E+04 | 2.50E+04 | 25028.7513 | -3 |
| oxFc Glycosylation G0 N (1) | 6.83 | 25085.852 | 1.05E+05 | 2.51E+04 | 25085.8026 | 2 |
| redFc_1 +Lysine C-TERM (1), Glycosylation Man5 N (1) | 6.83 | 25136.4719 | 4.78E+03 | 2.51E+04 | 25135.9028 | 22.6 |
| redFc_1 Glycosylation G1F-GlcNAc N (1) | 6.83 | 25194.6028 | 1.16E+04 | 2.52E+04 | 25194.9237 | -12.7 |
| redFc_1 Glycosylation G0F N (1) | 6.83 | 25236.9258 | 5.04E+03 | 2.52E+04 | 25235.9756 | 37.7 |
| oxFc__2 Glycosylation G0F-GlcNAc N (1) | 7.12 | 25029.2525 | 2.38E+04 | 2.50E+04 | 25028.7513 | 20 |
| oxFc__2 +Lysine C-TERM (1), Glycosylation G0 N (1) | 7.12 | 25213.2062 | 7.11E+04 | 2.52E+04 | 25213.9749 | -30.5 |
| redFc_2 +Lysine C-TERM (1), Glycosylation G1F N (1) | 7.12 | 25525.5292 | 2.54E+04 | 2.55E+04 | 25526.2885 | -29.7 |
| oxFc__2 Glycosylation G0F N (1) | 7.12 | 25232.0875 | 1.11E+06 | 2.52E+04 | 25231.9438 | 5.7 |
| redFc_2 Glycosylation G1 N (1) | 7.12 | 25252.3904 | 1.88E+05 | 2.53E+04 | 25251.975 | 16.4 |
| redFc_2 +Lysine C-TERM (1), Glycosylation G1F-GlcNAc N (1) | 7.12 | 25322.8147 | 4.36E+04 | 2.53E+04 | 25323.096 | -11.1 |
| redFc_2 Glycosylation G2F N (1) | 7.12 | 25560.3729 | 2.40E+04 | 2.56E+04 | 25560.2568 | 4.5 |
| oxFc Glycosylation G0F N (1) | 7.31 | 25231.1779 | 2.28E+04 | 2.52E+04 | 25231.9438 | -30.4 |
| oxFc Glycosylation G1 N (1) | 7.31 | 25248.4056 | 7.32E+04 | 2.52E+04 | 25247.9432 | 18.3 |
| oxFc +Lysine C-TERM (1), Glycosylation G0F N (1) | 7.31 | 25359.841 | 7.39E+03 | 2.54E+04 | 25360.1161 | -10.8 |
| oxFc Glycosylation G1F N (1) | 7.31 | 25393.4062 | 2.17E+04 | 2.54E+04 | 25394.0844 | -26.7 |
| oxFc Glycosylation G1F N (1) | 7.63 | 25393.9373 | 2.72E+05 | 2.54E+04 | 25394.0844 | -5.8 |
| oxFc Glycosylation G2 N (1) | 7.63 | 25409.2256 | 2.35E+04 | 2.54E+04 | 25410.0838 | -33.8 |
| oxFc Glycosylation G0F-GlcNAc N (1) | 7.76 | 25029.4797 | 5.22E+03 | 2.50E+04 | 25028.7513 | 29.1 |
| oxFc Glycosylation G1F N (1) | 7.76 | 25393.8579 | 1.13E+05 | 2.54E+04 | 25394.0844 | -8.9 |
| oxFc Glycosylation G2 N (1) | 7.76 | 25410.042 | 1.92E+04 | 2.54E+04 | 25410.0838 | -1.6 |
| oxFc Glycosylation G2F N (1) | 8.27 | 25556.1138 | 2.23E+04 | 2.56E+04 | 25556.225 | -4.4 |
| redFc Glycosylation G2F+SA N (1) | 8.75 | 25850.7718 | 4.14E+03 | 2.59E+04 | 25851.5114 | -28.6 |
| redFc Glycosylation G2F N (1) | 9.28 | 25561.1395 | 3.26E+03 | 2.56E+04 | 25560.2568 | 34.5 |

**Supplementary Figure 1** Linearity assessment of the sub-unit glycan method.

**Supplementary Table 3** Accuracy and Precision assessment of the sub-unit glycan method

|  | **Accuracy** | | | **Precision** | | |
| --- | --- | --- | --- | --- | --- | --- |
| **Major Glycans** | **% Relative Distribution (Area)** | **Min % Recovery** | **Max % Recovery** | **%RSD** | **Analyst 1 %RSD** | **Analyst 2 %RSD** |
| **G0** | 9.9 | 91 | 105 | 6 | 6 | 3 |
| **G0-GlcNAc** | 2.3 | 96 | 117 | 14 | 14 | 9 |
| **G0F** | 43.4 | 91 | 103 | 5 | 3 | 2 |
| **G1** | 17.5 | 90 | 119 | 8 | 8 | 7 |
| **G1F** | 24.2 | 87 | 107 | 4 | 3 | 4 |
| **G2F** | 0.9 | 82 | 118 | 42 | 30 | 13 |

**Supplementary Table 4** At-Line analysis of Glycan Distribution in Control Perturbated (no response) and Perturbated with Adaptive Control Bioreactor runs over time.

| **Run** | **Culture Day** | **G0F** | **G0-GlcNAc** | **G0** | **G1** | **G1F** | **G2F** | **G2F+SA** | **G2F+2SA** |
| --- | --- | --- | --- | --- | --- | --- | --- | --- | --- |
| Pertubated | Day 5 | 70.8% | <0.1% | 4.1% | <0.1% | 25.1% | <0.1% | <0.1% | <0.1% |
| Pertubated | Day 6 | 65.1% | <0.1% | 5.6% | <0.1% | 27.4% | 1.5% | 0.3% | <0.1% |
| Pertubated | *Day 7 | -- | -- | -- | -- | -- | -- | -- | -- |
| Pertubated | Day 8 | 68.9% | 0.7% | 10.8% | <0.1% | 18.7% | 0.6% | 0.3% | <0.1% |
| Pertubated | Day 9 | 66.1% | 0.9% | 11.1% | <0.1% | 21.3% | 0.6% | <0.1% | <0.1% |
| Pertubated | Day 10 | 64.6% | 1.1% | 11.3% | <0.1% | 22.6% | 0.5% | <0.1% | <0.1% |
| Pertubated | Day 11 | 63.0% | 1.7% | 12.8% | <0.1% | 22.5% | <0.1% | <0.1% | <0.1% |
| Pertubated | Day 12 | 59.8% | <0.1% | 20.7% | <0.1% | 19.5% | <0.1% | <0.1% | <0.1% |
| Pertubated | Day 13 | 66.1% | 1.3% | 15.9% | 3.1% | 13.6% | <0.1% | <0.1% | <0.1% |
| Pertubated | Day 14 | 75.2% | 1.1% | 13.2% | <0.1% | 10.5% | <0.1% | <0.1% | <0.1% |
| Adaptive Control | Day 5 | 70.5% | 0.2% | 6.1% | <0.1% | 21.8% | 1.5% | <0.1% | <0.1% |
| Adaptive Control | Day 6 | 66.2% | <0.1% | 5.2% | 2.6% | 24.1% | 1.4% | <0.1% | 0.5% |
| Adaptive Control | Day 7 | 71.7% | <0.1% | 6.1% | <0.1% | 21.1% | 1.0% | <0.1% | <0.1% |
| Adaptive Control | Day 8 | 72.0% | <0.1% | 8.0% | 3.1% | 16.1% | 0.8% | <0.1% | <0.1% |
| Adaptive Control | Day 9 | 69.0% | 1.2% | 14.4% | 3.1% | 12.4% | <0.1% | <0.1% | <0.1% |
| Adaptive Control | Day 10 | 66.5% | 0.9% | 13.4% | 4.2% | 14.2% | 0.8% | <0.1% | <0.1% |
| Adaptive Control | Day 11 | 65.0% | 1.9% | 11.5% | 4.9% | 16.7% | <0.1% | <0.1% | <0.1% |
| Adaptive Control | Day 12 | 62.3% | 2.6% | 14.3% | 5.8% | 15.0% | <0.1% | <0.1% | <0.1% |
| Adaptive Control | Day 13 | 58.1% | 2.9% | 14.6% | 6.8% | 16.6% | 1.0% | <0.1% | <0.1% |
| Adaptive Control | Day 14 | 57.8% | 3.5% | 14.1% | 7.8% | 16.8% | <0.1% | <0.1% | <0.1% |

*Day 7 sample for the perturbated run is missing due to an error on the cell removal system resulting in no sample being pulled from the bioreactor at that time.
